# Environmental and Genetic Determinants of Colony Morphology in Yeast

**DOI:** 10.1371/journal.pgen.1000823

**Published:** 2010-01-22

**Authors:** Joshua A. Granek, Paul M. Magwene

**Affiliations:** Department of Biology and Center for Systems Biology, Duke University, Durham, North Carolina, United States of America; Stanford University School of Medicine, United States of America

## Abstract

Nutrient stresses trigger a variety of developmental switches in the budding yeast *Saccharomyces cerevisiae*. One of the least understood of such responses is the development of complex colony morphology, characterized by intricate, organized, and strain-specific patterns of colony growth and architecture. The genetic bases of this phenotype and the key environmental signals involved in its induction have heretofore remained poorly understood. By surveying multiple strain backgrounds and a large number of growth conditions, we show that limitation for fermentable carbon sources coupled with a rich nitrogen source is the primary trigger for the colony morphology response in budding yeast. Using knockout mutants and transposon-mediated mutagenesis, we demonstrate that two key signaling networks regulating this response are the filamentous growth MAP kinase cascade and the Ras-cAMP-PKA pathway. We further show synergistic epistasis between Rim15, a kinase involved in integration of nutrient signals, and other genes in these pathways. Ploidy, mating-type, and genotype-by-environment interactions also appear to play a role in the controlling colony morphology. Our study highlights the high degree of network reuse in this model eukaryote; yeast use the same core signaling pathways in multiple contexts to integrate information about environmental and physiological states and generate diverse developmental outputs.

## Author Summary

Baker's yeast forms smooth round colonies when grown in favorable conditions. When starved for one or more nutrients, yeast can alter its growth pattern to produce complex structures consisting of numerous interacting cells. One mode of growth, the colony morphology response, produces visually striking, lacy colony architectures. We describe both conditions that induce this morphology and also genes and pathways that are required for the response. We demonstrate that low levels of carbon combined with abundant nitrogen trigger complex colony formation. Using a candidate gene approach coupled with genome-wide mutagenesis, we identified genes involved in the production of complex colony morphology. Many of these genes are components of either a MAP kinase cascade or the Ras-cAMP-PKA pathway, two well-studied signaling pathways that are conserved across eukaryotic organisms. Yeast use these pathways to mediate cellular responses to changes in their environment. We observe shared characteristics between complex colonies and biofilms, which are organized communities of microorganisms with relevance to human health and human infrastructure, making colony morphology a candidate model for understanding how microorganisms interact to form complex structures.

## Introduction

Baker's yeast, *Saccharomyces cerevisiae*, is most often described as a simple, unicellular organism. Despite this perception, *S. cerevisiae* displays a surprising array of behaviors, many of them involving complex interactions between cells. Under nutrient rich conditions, *S. cerevisiae* grows via “yeast form,” mitotic growth, rapidly dividing and forming smooth, round colonies on solid media. Limitation of one or more key nutrients can trigger a variety of developmental responses. For example, nitrogen starvation of diploid cells induces pseudohyphal growth, which is characterized by elongated cells, agar invasion and unipolar budding, where mother and daughter cells remain attached [1]–[3]. Haploid invasive growth, a similar behavior, is observed in haploid cells grown under dextrose limitation [4], or in the presence of various alcohols [5]–[7]. Nitrogen starvation combined with a non-fermentable carbon source induces sporulation and meiosis [8]–[11].

A number of yeast developmental responses result in multicellular structures. For example, biofilm mat formation is induced by growth on solid media with low agar and dextrose concentrations [12]. The combination of plating on hard agar followed by UV irradiation has been shown to trigger the growth of multicellular, macroscopic stalks [13]. Cell-cell adhesion is a necessary component of these responses and is induced by several different stresses including carbon and nitrogen starvation and changes in ethanol concentration and pH [14]. Recent work suggests a quorum sensing mechanism in *S. cerevisiae* based on the autostimulatory aromatic alcohols phenylethanol and tryptophol. This quorum sensing mechanism has been shown to enhance filamentous growth, and presumably contributes to other developmental responses as well [15].

**Figure 1:**
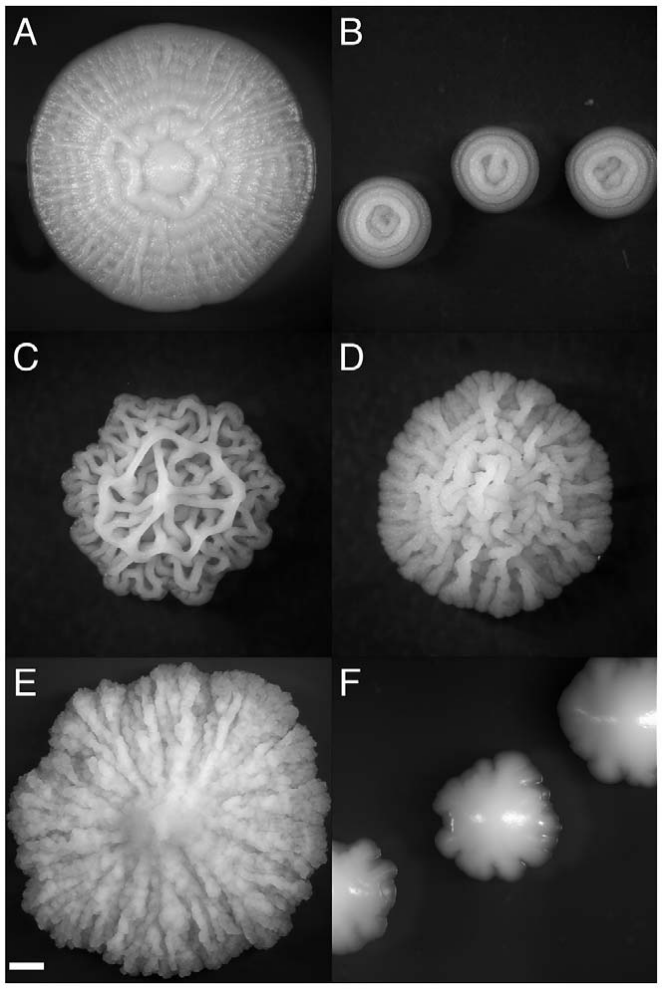
Strain-specific variation in complex colony morphotype. Characteristic CCM morphotypes fall into several categories (A) spokes (with weak concentric rings in this case)(OS17, YEPLD, day 6), (B) concentric rings (YJM224, 0.25% dextrose YEPD, day 3) (C) lacy (YJM311 on YEPLD, day 6), (D) coralline (NKY292, 1% dextrose YEPD, day 6), (E) mountainous (PMY348, 4% agar YEPD, day 6), (F) irregular (BY4743, YEPSucrose, day 5). Scale bar is 1 mm.

In addition to the developmental responses described above, *S. cerevisiae* can form colonies consisting of complex, organized, macroscopic structures (Figure 1). We refer to the induction of this phenotype as the “colony morphology response.” The determinants and function of the colony morphology response are poorly understood in yeast. Complex colonies produce an extensive extracellular matrix that is absent from simple colonies [16], and it has been proposed that complex colonies help protect yeast cells against a hostile environment [17]. It has been observed that starvation results in the reorganization of yeast colonies at the cellular level [18], and there is evidence that budding patterns and distributions of cell shape are different in complex colonies than simple colonies [19]. Microarray expression analysis comparing a strain with a complex colony phenotype and a strain with smooth colonies, derived from the first by passaging on rich media, found numerous differences in their transcriptional profiles [16]. However, it is impossible to tell which of these changes are cause, which are effect, and which are unrelated to the colony morphology response.

The colony morphology response is a promising system for the study of simple multicellular developmental processes because it involves cell-cell communication, cellular differentiation and specialization, and cell-adhesion. While the mechanisms involved in the development of complex yeast colonies are unlikely to be evolutionarily related to the developmental pathways regulating multicellularity in metazoans, *S. cerevisiae* offers the opportunity to explore the principles underlying multicellular differentiation in an extremely tractable model system. As a “facultative” multicellular behavior of a unicellular organism, complex colony formation raises interesting questions of cooperative behavior and the repeated evolution of multicellularity across the tree of life [20]. Similar colony morphologies are observed in many undomesticated bacteria [21]. This gross similarity at the macroscopic scale begs the question of whether such structures represent convergent, adaptive solutions that microbial lineages have evolved to deal with similar environmental challenges.

In this report, we define key environmental and genetic determinants of complex colony morphology in *S. cerevisiae*. By studying the phenotypes of a genetically diverse panel of *S. cerevisiae* isolates under a large number of growth conditions we have determined that fermentable carbon source limitation plus an abundant nitrogen source are the key nutritional signals for inducing complex colony morphology. We show that the complex colony response requires the filamentous growth MAP kinase (FG MAPK) cascade and Ras-cAMP-PKA signaling and that mutations at the RIM15 locus exhibit synergistic epistasis with components of these pathways. We also demonstrate that ploidy and mating type quantitatively contribute to the intensity of colony morphology and that genotype-by-environment effects are common for this trait.

## Results

### Carbon Source Limitation Plus Nitrogen Abundance Induces Complex Colony Morphology

**Figure 2:**
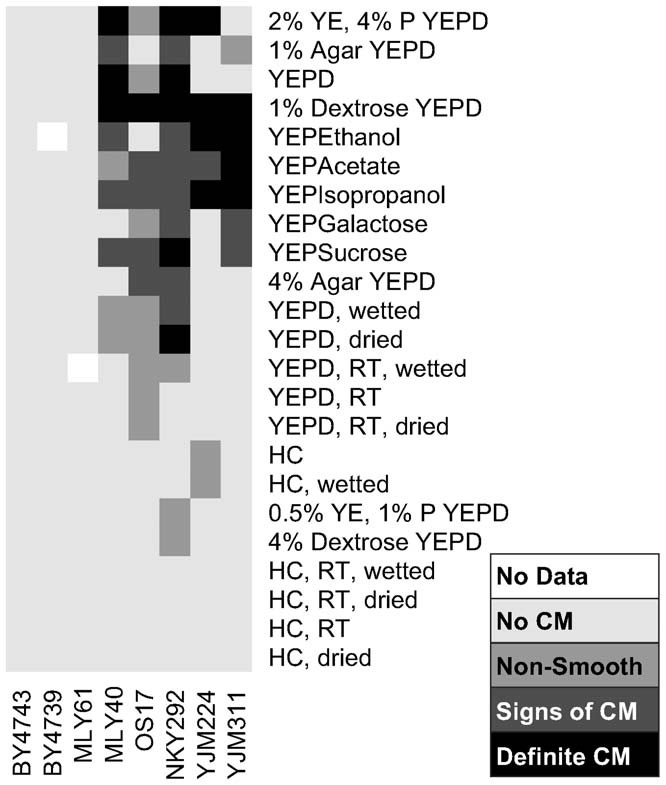
Extent of complex colony morphology under a variety of growth conditions. Summary of colony morphology phenotypes for eight strains under thirty-four growth conditions. Darker colors from light gray to black indicate increasing colony morphology response. 0.5% YE, 1% P YEPD: 0.5% yeast extract, 1% peptone YEPD. RT: grown at room temperature, dried: media partially dried in oven, wetted: media to which 400 µl H2O was added after plates set, HC: Hartwell's Complete media.

We studied eight strains of *S. cerevisiae* (BY4743, BY4739, MLY40α, MLY61a/α, YJM224, YJM311, OS17, NKY292) under a variety of growth conditions (Table S1) in order to determine the most important environmental triggers for complex colony morphology (CCM). This strain panel was chosen to include common laboratory strain backgrounds - S288c (BY4743 [diploid] and BY4749 [haploid]), SK1 (OS17 [diploid] and NKY292 [haploid]), and Σ1278b (MLY61a/α [diploid] and MLY40α [haploid]) - as well as a distillery strain (YJM224 [diploid]) and a clinical isolate (YJM311 [diploid]). Σ1278b and SK1 are standard backgrounds for studying yeast development (sporulation in SK1, filamentous growth in Σ1278b) and their inclusion here facilitates comparisons between developmental processes. We varied the conditions of growth along five major axes: carbon source type and concentration, non-carbon nutrient concentration, media water content, media hardness (agar content), and temperature. Growth was monitored daily for six days, and each plate was scored for colony morphology (Figure 2). This survey showed that induction of colony morphology is primarily carbon source dependent, with the strongest effects induced by reduced dextrose (1% dextrose w/v) and non-fermentable carbon sources (isopropanol, ethanol, acetate). Increasing dextrose concentration (4% Dextrose YEPD) inhibits the colony morphology response, providing further evidence that carbon source limitation is a primary trigger for CCM. In contrast, media water content and hardness had little if any effect on CCM induction. The only obvious effect of temperature was slow growth at lower temperature, which prolonged the time course of colony development.

**Figure 3:**
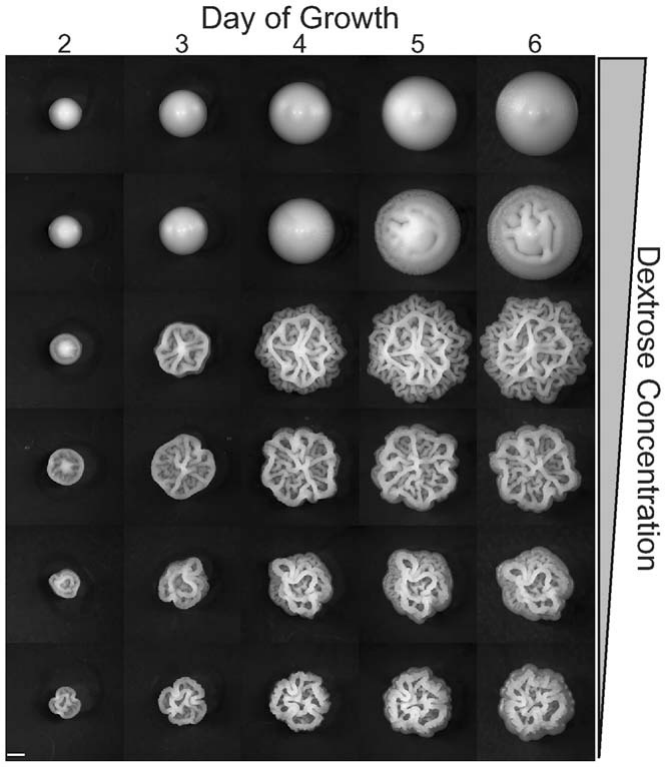
Colony morphology as a function of time and dextrose concentration. Colonies of YJM311 were grown on YEPD with dextrose concentrations ranging from 2% to 1/16% in two-fold steps, and imaged daily for six days. Lower dextrose concentrations more strongly induce the colony morphology response. Scale bar is 1 mm.

We further investigated the impact of carbon availability on CCM induction by growing the same strains on YEPD plates containing a range of dextrose concentrations, from 2% (standard YEPD) to 0.0625% (Figure 3). We observed two major trends in this experiment. First, the lowest concentrations of dextrose caused the fastest induction of CCM. On lower dextrose concentrations CCM is observable as early as day two for some strains (Figure 3). Second, there is strain-to-strain variation in dextrose sensitivity. By day six most CCM competent strains exhibit the phenotype on 1% dextrose (MLY40α, OS17, NKY292, and YJM311) and even weakly on 2% dextrose (NKY292), while others (YJM224) required a dextrose concentration of 0.5% or less to induce the colony morphology response. At the lower end of the dextrose concentrations tested, colonies were smaller at each time point, presumably because they exhausted all available carbon, or the low levels of carbon induced growth regulation. At the lowest dextrose concentrations some strains failed to demonstrate the strain specific colony morphotypes observed at intermediate concentrations, likely because of growth limitations.

**Figure 4:**
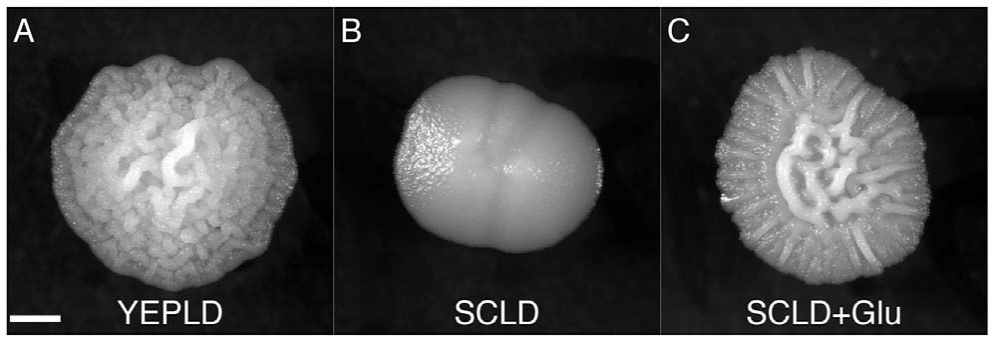
A rich nitrogen source is required for induction of the colony morphology response. The complex colony response is induced in PMY574 by growth on (A) YEPLD, but not on (B) SCLD. Growth on (C) SCLD supplemented with glutamate (SCLD+Glu) recovers the complex colony response. Scale bar is 1 mm.

Other nutrients also play a role in the complex colony response. Reducing yeast extract and peptone to half of the normal YEPD levels inhibits complex morphology, and doubling these nutrients induces it (Figure 2). We suspected that nitrogen might be the key nutrient causing this effect. To test this hypothesis we assayed colony morphology on synthetic media (SC) with and without the addition of glutamate, a preferred nitrogen source [22],[23]. None of the strains tested exhibited complex morphologies on 0.5% Dextrose SC (SCLD), but when the synthetic media is supplemented with 50mM glutamate (SCLD+Glu), some strains developed complex morphologies like those observed on YEPLD, while others developed intermediate morphologies (Figure 4 and Figure S1). The most glucose sensitive of the strains in our survey (YJM224) displayed only simple morphology on the glutamate supplemented SCLD media. Higher levels of glutamate (200mM) resulted in little if any additional changes in colony morphology (data not shown).

### Identification of Genes Involved in Complex Colony Morphology

Because there are significant pleiotropic interactions between developmental pathways in yeast [24] we hypothesized that the signaling and regulatory pathways controlling the colony morphology response would show some degree of overlap with those regulating other developmental responses, such as pseudohyphal growth, haploid invasive growth, and sporulation. To test this, we assayed colony morphology phenotypes in a panel of knockout mutants of genes known to be involved in developmental processes. This panel consisted of over 150 strains representing more than 50 different gene knockouts in *MATa*, *MATα*, and *MATa*/*MATα* strains of two lineages of the Σ1278b background. Wild-type diploid Σ1278b shows simple colony morphology in our assays while haploid Σ1278b shows strong complex morphology (see section on ploidy below). We identified thirteen haploid loss-of-CCM mutants and four diploid gain-of-CCM mutants (Table 1). We found that some gene-knockouts behaved differently in the different lineages of the Σ1278b background. For example, the *tpk3Δ/ tpk3Δ* diploid mutants exhibit a gain of CCM in the “Heitman” Σ1278b background [25], but not in the Sigma2000 background [26]. This variation is likely due to small genetic differences between these strains (see below) resulting from distinct histories of strain construction [27]. In some cases we observed differences in the phenotypes of gene-knockouts between *MATa* and *MATα* strains (Table 1, Figure S2 and Figure S3). In addition to the four diploid mutants listed in Table 1, we observed that a *hog1Δ*/*hog1Δ* mutant had a gain-of-CCM when grown on YEPD, YEPLD, YEPHD, and YEPEthanol (Figure S4). This pattern of induction suggests that crosstalk between various signal transduction pathways, which has been observed to cause inappropriate responses to environmental signals [28]–[30], can also induce complex colony morphology as well.

**Table 1:** Mutations with colony morphology phenotypes.

Gene Knockout	Haploid Mutant Phenotype	Diploid Mutant Phenotype	Notes
*cln1Δ*	−	=	a
*flo11Δ*	−	=	ce
*gln3Δ*	−	=	ce
*ras2Δ*	−	=	c
*ste11Δ*	−		c
*ste12Δ*	−	=	ace
*ste20Δ*	−	=	ce
*ste7Δ*	−	=	ce
*tec1Δ*	−	=	c
*tpk1Δ*, *tpk2Δ*	−	=	ce
*tpk2Δ*, *tpk3Δ*	−	=	ace
*ira2Δ*	=	+	ac
*tec1Δ*, *dig1Δ*, *dig2Δ*		+	e
*tpk1Δ*, *tpk3Δ*	=	+	ce
*tpk3Δ*	=	+	cd
*tpk2Δ*	−	=	a
*mga1Δ*	−	=	ab
*elp4Δ*	−		c
*pet122Δ*	−		c
*rgt1Δ*	−		c
*rrt12Δ*	−		ac
*rsc1Δ*	−		c
*trm9Δ*	−		c
*yta7Δ*	−		c

“Mutant phenotype” indicates whether the gene knockout strain has a significant change in colony morphology relative to WT grown on YEPLD. “−” indicates a significant decrease in CCM, “+” indicates a significant increase in CCM, “ = ” indicates no significant change. Blanks indicate mutants not tested or giving inconsistent results.

*Notes:* (a) Phenotype difference between a and α; (b) Phenotype difference between diploid backgrounds; (c) Haploid tested in only one strain background; (d) Phenotype difference between diploid backgrounds; (e) Diploid tested in only one strain background.

In order to gain a more comprehensive understanding of the genes and pathways affecting colony morphology phenotypes, we carried out a transposon mutagenesis screen using the mTn7-mutagenized genome library created by Kumar et al [31]. This screen identified seven additional genes exhibiting loss-of-CCM mutant phenotypes: YTA7, RSC1, RGT1, RRT12, TRM9, ELP4, and PET122. Most of these genes have been previously described as affecting developmental pathways. Both ELP4 and TRM9 are members of the tRNA modification elongator complex. Other members of the elongator complex are required for filamentous growth and elp2Δ mutants show reduced biofilm mat formation [32]. Fischer et al showed that deletion of RSC1 impairs FLO11 expression and hence leads to a loss of invasive and pseudohyphal growth [33]. YTA7 is involved in chromatin silencing and maintains a barrier between heterochromatin and euchromatin upstream of the silent HMR locus [34]. In other screens, YTA7 mutants have been found to have a loss of “fluffy” colony morphology [35] and decreased filamentous growth [36]. RGT1 encodes a glucose responsive transcription factor and mutations in this gene are known to cause sporulation defects, though this may result from decreased cell size in these mutants [37]. RRT12 (OSW3) encodes a protein involved in the formation of a protective dityrosine coat required for spore wall assembly [38].

### Mutations in RIM15 Exhibit Epistasis

**Figure 5:**
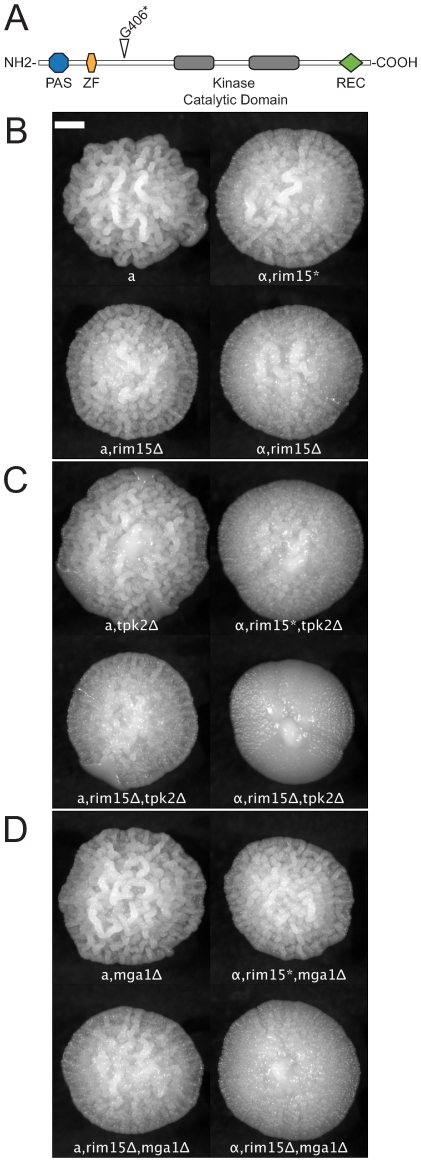
Synergistic epistatic effects of RIM15 mutations. (A) The domain structure of Rim15p [39]. The nonsense mutation at residue 406, identified in the strain MLY40α (Σ1278b, Heitman lineage) is indicated by the open triangle. *rim15*, *tpk2Δ*, and *mga1Δ* mutants show weak or no effect on colony morphology by themselves but the double mutants exhibit a synergistic interaction. (B) *rim15Δ* and rim15* (opal allele, 1216G>T) mutations in *MATa* and *MATα* backgrounds; (C) *tpk2Δ* single mutant and *rim15*, *tpk2Δ* double mutants in *MATa* and *MATα* backgrounds. (D) *mga1Δ* single mutant and *rim15*, *mga1Δ* double mutants in *MATa* and *MATα* backgrounds. Scale bar is 1 mm.

As described above, we observed phenotypic differences among knockout mutants in different lineages of the Σ1278b background, and in some cases we noted differences between *MATa* and *MATα* strains, particularly in the “Heitman” Σ1278b background. Because this variation was consistent between experimental replicates, we reasoned that the phenotypic variation we observed was due to mutations that accumulated in each lineage during laboratory domestication. We used SNP calls from high-throughput sequencing data (Magwene, in prep.) to identify heterozygous sites in the diploid strain MLY61a/α, created from a cross of MLY40α and MLY41a. We then predicted which of these sites were heterozygous for premature stop codons (relative to the predicted peptide sequences of the reference strain S288c). Among the heterozygous sites we identified was a nonsense mutation in RIM15, a G >T transversion at position 1216 that converts a Gly codon to an opal stop codon (*rim15:1216G>T*). Rim15p is a protein kinase shown to play a key role in mediating developmental responses to nutrient conditions [39],[40]. The wild-type RIM15 encodes a 1770aa long protein. The *rim15:1216G>T* allele encodes a truncated protein with a predicted length of 406aa, which includes two putative functional domains (PAS and zinc-finger) [39], but not the kinase domain (Figure 5A). We confirmed the presence of two distinct alleles in the Heitman lineage by sequencing a 312bp portion of RIM15 covering the polymorphic site, from MLY61a/α, MLY40α, MLY41a, and G85 (Sigma2000). This confirmed that MLY61a/α was heterozygous, MLY40α, bore the predicted *rim15:1216G>T* allele, and MLY41a encodes the full length (wild-type) RIM15. G85 is homozygous for the wild-type allele.

The *MATα* strain, MLY40α, reproducibly develops a subtly weaker form of the complex colony phenotype than does the *MATa* strain, MLY41a (Figure 5B, top). We predicted that this was due to a partial or complete loss of Rim15p function. To test this we compared the colony morphology of XPY90a and XPY90α (*rim15Δ::HygB* derivatives of MLY41a and MLY40α respectively) [41] with that of MLY41a and MLY40α. As predicted, the *rim15Δ* mutants (Figure 5B, bottom) exhibited a colony morphology phenotype very similar to that of MLY40α and decreased relative to MLY41a (compare top and bottom rows of Figure 5B). We also noted differences between *MATa* and *MATα* strains for several of the deletion mutants we tested (Figure 5C and 5D). We predicted that these differences reflected epistatic interactions between RIM15 and the gene knocked out, such that a gene deleted in MLY41a was the expected single knockout, whereas the same deletion in MLY40α was effectively a double-mutant with *rim15:1216G>T*. To test this we crossed XPY5a (*MATa*, *tpk2Δ*) with XPY90α (*MATα*, *rim15Δ*) and MLY179α (*MATα*, *mga1Δ*) with XPY90a (*MATa*, *rim15Δ*) and analyzed how colony morphology segregated in tetrads relative to mating type and the gene deletions. The results of these crosses indicate the following: 1) both mutations at the RIM15 locus (*rim15Δ* and *rim15:1216G>T*) interact epistatically with mutations at the TPK2 and MGA1 loci such that the degree of colony morphology loss is greater than the sum of the single mutants (*rim15Δ*, *tpk2Δ* < *rim15Δ* or *tpk2Δ* and *rim15Δ*, *mga1Δ* < *rim15Δ* or *mga1Δ*); 2) the *rim15:1216G>T* allele may maintain some functionality because the degree of CCM reduction observed in mutants with this background are typically milder than those for comparable mutants in the *rim15Δ* background and; 3) there is still an effect of mating type on the degree of colony morphology independent of the RIM15 locus. These findings are illustrated in Figure 5B–5D. Results of the crosses are thus consistent with a model of synergistic epistatic interaction between RIM15 and other genes involved in colony morphology.

### Role of Ploidy in Colony Morphology

**Figure 6:**
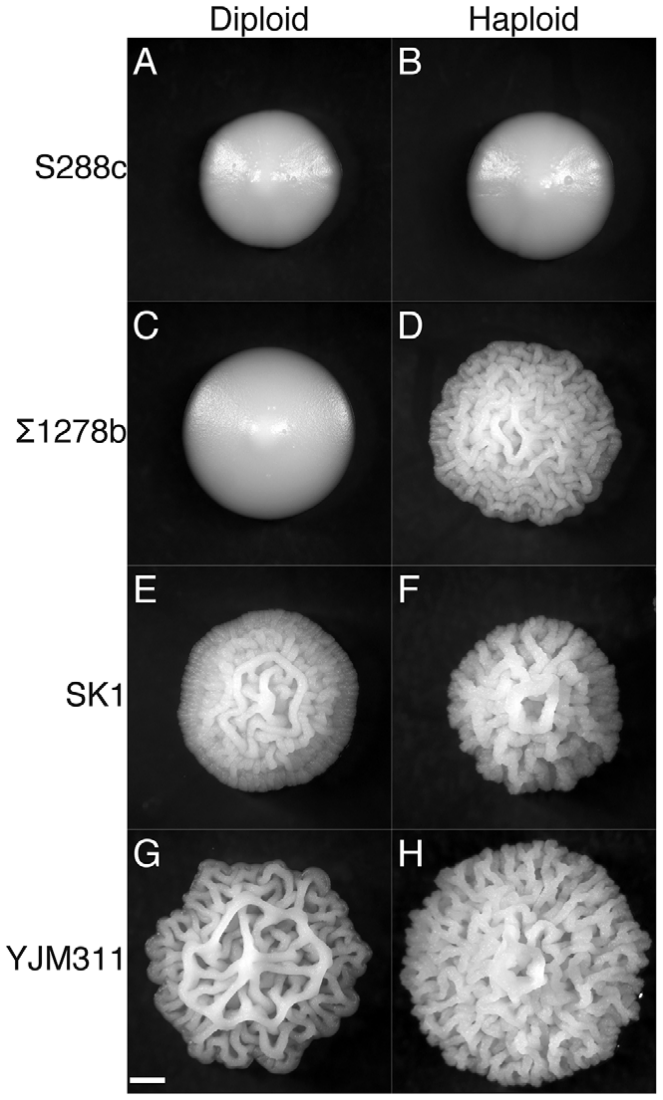
Ploidy affects colony morphotypes and strength of induction of the colony morphology response. The S288c background [(A) BY4743, (B) BY4739] forms only simple colonies. Σ1278b diploid colonies [(C) MLY61a/α] are simple, while the haploid colonies [(D) MLY40α] are complex. Both SK1 [(E) OS17] and YJM311 [(F) NKY292] diploids form complex colonies, but the morphotypes are distinct from haploids [(G) YJM311, (H) PMY556] in these backgrounds. Day 6 of growth on YEPLD. Scale bar is 1 mm.

In addition to nutritional determinants, we observed a role for ploidy in the colony morphology response. Several strains that have simple or mild colony morphologies as diploids (MLY61a/α and OS17) exhibit strong colony phenotypes as haploids (MLY40α and NKY292) (contrast Figure 6C and 6E with Figure 6D and 6F). To further explore the colony morphology differences between isogenic haploids and diploids, we constructed haploid derivatives of a clinical isolate (YJM311) that exhibits a strong CCM phenotype as a diploid. We observed variation in colony morphology among the haploid derivatives of this strain, presumably due to allelic heterozygosity in the parental strain, but many displayed a morphology similar to that found in other haploid strains (compare Figure 6H with Figure 6D and 6F).

In order to confirm the role of ploidy in the colony morphology response we tested a set of isogenic haploid, diploid, triploid, and tetraploid strains [42] for colony morphology phenotypes in the Σ1278b.. We found an inverse correlation between ploidy and colony morphology; strains with ploidy of 2N and greater showed mild or no signs of complex colony morphology (Figure S5). Here as well mating type has a weak but noticeable affect on colony morphology independent of ploidy. The diploids heterozygous at the MAT locus (the normal state for diploids; Figure S5E) have simple morphology, while those homozygous for MAT have colonies that are somewhat elaborated (Figure S5B).

### Genotype-by-Environment Interactions

During our survey of growth conditions, we observed that colony morphology exhibits genotype-by-environment (G×E) effects. To provide a framework for study of G×E interactions we defined six morphotype classes: spokes (with weak concentric rings in this case) (Figure 1A), concentric rings (Figure 1B), lacy (Figure 1C), coralline (similar to lacy, but the cable-like structures are more angular, and tend to have more height) (Figure 1D), mountainous (possibly a variation on spokes) (Figure 1E), and irregular (which includes a wide range of forms that have no obvious regularity) (Figure 1F). For example, YJM311 grown on YEPLD media has a “lacy” morphotype (Figure 1C). The same strain grown on YEPEthanol, YEPIsopropanol, or YEPAcetate (Figure S6A, S6B, S6C) has a morphology that closely resembles a tangle of string (a variation of the lacy morphotype). On galactose, sucrose, and 1% agar YEPD the same strain exhibits the spoke morphotype, although each media induces a distinct version of the spoke morphotype (Figure S6D, S6E, S6F).

### Survey of Colony Morphology Frequency and Types

Having identified the key signals for the colony morphology response, we expanded our survey to include all 35 *S. cerevisiae* strains from the *Saccharomyces* Genome Resequencing Project (SGRP; [43]). Our goal was to determine the prevalence and diversity of complex colony morphologies and to identify strains of interest for future work. Of these thirty-five strains, by day six of growth, thirteen exhibited non-smooth or stronger colonies (anything beyond a smooth, shiny colony) on at least one media type. For most of these, this was simply a bumpy or textured colony surface, but six of these thirteen had at least “signs of CCM” (score of two or greater) (Figure S7).

## Discussion

### Carbon and Nitrogen Availability Regulate Complex Colony Morphology

In common with other developmental switches in yeast, the complex colony morphology response is induced by nutritional signals. Fermentable carbon source limitation coupled with an abundant nitrogen source appears to be the key trigger.

**Figure 7:**
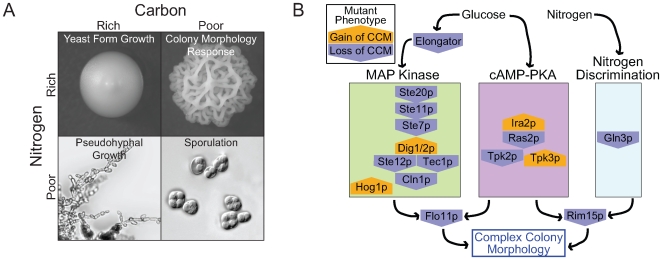
The role of nutrients in complex colony morphology and the underlying genetic network. (A) The quantity and quality of available carbon and nitrogen controls developmental responses in *S. cerevisiae*. (B) Many genes involved in the colony morphology response play roles in the MAPK, cAMP-PKA, or Nitrogen Discrimination pathways. These pathways are responsible for sensing glucose, nitrogen, pheromone, and osmolarity. Mutant phenotypes include both gain (orange pentagon pointing up) and loss (blue pentagon pointing down) of complex colony morphology (relative to WT).

Taking our results together with information on other developmental responses sheds light on how *S. cerevisiae* responds to variable nutritional environments (Figure 7A). Haploid invasive growth, like complex colony morphology, is induced by dextrose limitation [4]. What seems to distinguish the two is the availability of other nutrients, particularly nitrogen. CCM competent strains grown on low dextrose synthetic media do not generally exhibit complex morphology. However, supplementing this synthetic media with glutamate is sufficient to induce the colony morphology response in most competent strains. In contrast, nitrogen availability seems to have little effect on haploid invasive growth [4].

Our findings also suggest a link between complex colony morphology and *S. cerevisiae* biofilm formation [12]. Like complex colony morphology, reduced dextrose is a trigger for biofilm development, and biofilms exhibit gross structural features resembling some of the colony structures we have observed [12],[44]. Cellular level organizational changes observed in starving colonies [18] might help explain how starvation signals result in macroscopic changes in both colony and biofilm structure.

### MAP Kinase and Ras-cAMP-PKA Pathways Are Required for Complex Colony Morphology

The emerging picture of yeast development suggests that *S. cerevisiae* uses the core elements of two key signaling pathways, a MAP kinase cascade and a Ras-cAMP-PKA pathway, in multiple contexts [1],[3],[45]. The colony morphology phenotypes we observed in knockout strains implicate both of these pathways as playing key roles in regulating colony architecture (Figure 7B).

### MAP Kinase Signaling

The filamentous growth/mating MAPK cascade (consisting, in part, of the kinases Ste20p, Ste11p, and Ste7p) regulates mating, filamentous and invasive growth, and cell wall integrity, in response to pheromone, nutrient limitation and osmolar stress respectively [46]. The mating and filamentous growth pathways both involve the transcription factor Ste12p, which induces expression of mating genes by binding pheromone response elements (PREs), and dimerizes with Tec1p to bind filamentous response elements (FREs) in the promoters of filamentation genes. Dig1p and Dig2p inhibit activation by Ste12p at PREs and by the Ste12p/Tec1p heterodimer at FREs [47].

Because multiple signals flow through the same core kinases of the MAPK cascade, several mechanisms are employed to prevent incorrect output. Knocking out genes in the cascade can disrupt this “insulation,” resulting in crosstalk between the pathways [28]–[30]. Such crosstalk is observed in *hog1Δ* mutants, which can be induced to mate by osmolar stress [29]. We observe similar crosstalk in the regulation of colony morphology. The diploid *hog1Δ/ hog1Δ* mutant exhibits colony morphology on low dextrose, high dextrose or alcohol containing media (Figure S4).

The crosstalk observed in MAPK cascade mutants complicates interpretation, but the loss of CCM in *ste20Δ*, *ste11Δ*, *ste7Δ*, *ste12Δ*, and *tec1Δ* mutants demonstrates that the MAPK cascade plays a key role in the regulation of colony morphology. We observed no gain of CCM in a diploid *ste12Δ/ste12Δ*, *dig1Δ/dig1Δ*, *dig2Δ/dig2Δ* triple mutant strain, but we did find a gain of CCM in the diploid *tec1Δ/tec1Δ*, *dig1Δ/dig1Δ*, *dig2Δ/dig2Δ* triple mutant. Our interpretation of this result is that when Dig1p/Dig2p repression of Ste12p is relieved, Ste12p is capable of activating a set of Tec1p independent targets, as has been show previously [48], and that this subset of targets affects colony morphology.

Our identification of ELP4 and TRM9 in the mutagenesis screen further argues for an important role of the MAPK cascade in regulating complex colony morphology. Abdullah and Cullen recently demonstrated a role for the elongator complex and other tRNA modification proteins in the MAPK dependent regulation of filamentous growth [32]. Elongator affects this pathway via starvation dependent induction of the signaling mucin gene *MSB2*, which interacts with Cdc42 to activate MAPK signaling [49]. Our independent identification of *elp4Δ* and *trm9Δ* mutants in this study adds to the evidence for a role for elongator and other tRNA modification complexes in regulating yeast development via the MAPK pathway.

### Ras-cAMP-PKA Signaling

In addition to the MAP kinase cascade, the colony morphology response also requires a functional Ras-cAMP-PKA pathway. Mutants that inhibit this pathway exhibit an attenuation of colony morphology, while those that up-regulate cAMP levels and/or PKA activation show an increased expression of complex morphology in diploid backgrounds.

A *ras2Δ* haploid mutant shows a loss of CCM consistent with similar decreases in biofilm formation and pseudohyphal growth observed for *ras2Δ* mutants [44],[50]. We also confirmed the observation of Halme et al. [51] that deletion mutants of IRA2 exhibit an increased colony morphology phenotype. Ira2p promotes Ras inactivation by stimulating GTPase activity, and treatment of cells with glucose destabilizes Ira2p, allowing active Ras proteins to induce cAMP production by adenylate cyclase [52].

There are three catalytic subunits of yeast PKA, Tpk1p, Tpk2p, and Tpk3p. Previous studies [41],[53],[54] have demonstrated distinct developmental and physiological roles for each of these subunits. For example, Tpk2p promotes filamentous growth and expression of Flo11p while Tpk1 and Tpk3p inhibit filamentous growth [41],[54]. Similar to these previous studies, we observed distinct effects of the PKA subunits on the colony morphology response. We found a loss of CCM in haploid *tpk2Δ* mutants as well as in *tpk1Δ*, *tpk2Δ* double mutants. The *tpk2Δ*, *tpk3Δ* double mutant showed a mild decrease in CCM. In diploids the *tpk3Δ/tpk3Δ* single mutant showed a background dependent increase in colony morphology. The *tpk1Δ/ tpk1Δ*, *tpk3Δ/tpk3Δ* double mutant also showed an increase in colony morphology. We did not observe a definite colony morphology phenotype in haploid or diploid TPK1 mutant strains or diploid TPK2 mutants. The opposite phenotypes of TPK2 and TPK3 mutants can be explained by a model put forth by Pan and Heitman [41] that suggests Tpk3p (and possibly Tpk1p) act in a negative feedback loop that attenuates cAMP levels. A candidate target for this feedback interaction via Tpk3p is the low-affinity phosphodiesterase Pde1p [55]. Our interpretation of this model and the mutants described above is that an active cAMP-PKA pathway is required for the development of complex colonies. Mutations that lead to a decrease in cAMP and/or PKA activation (*ras2Δ* and *tpk2Δ*) also decrease complex colony morphology and those that increase cAMP levels (*ira2Δ* and *tpk3Δ*) promote the development of complex colonies.

### Nitrogen Sensing

Given that a good nitrogen source seems to be a requirement for complex colony morphology, it is perhaps surprising that we observed a loss of CCM in a *gln3Δ* mutant. Gln3p is a transcriptional activator that activates “nitrogen starvation genes,” genes repressed by preferred nitrogen sources such as glutamate and ammonium. Under good nitrogen conditions, Gln3p is sequestered in the cytoplasm by Ure2p. Nitrogen deprivation leads to dissociation of Gln3p from Ure2p, Gln3 then localizes to the nucleus [23]. However, ours is not the first study to observe unintuitive results with respect to the effects of nitrogen catabolite repression pathway mutants on yeast development. Lorenz and Heitman [56] found that both a *gln3Δ/gln3Δ* mutant and a *ure2Δ/ure2Δ* mutant are defective in pseudohyphal growth. These results suggest that a balance between Ure2p and Gln3p may be necessary for appropriate response to nitrogen levels.

### Ploidy and Mating Type Affect Complex Colony Morphology

We find that ploidy has a major effect on colony morphology phenotypes. In some strains, this is manifested as a decrease in colony morphology in diploids relative to haploids; in others, there is simply a change in the stereotyped colony morphotype with ploidy. For example, colonies of Σ1278b haploids strains develop complex morphologies within six days, whereas diploid strains take much longer [19]. It has been proposed that this ploidy difference in colony morphology is linked to the ploidy specific expression of FLO11 [19],[42],[57]. The role of ploidy in the colony morphology response is another link between colony morphology and biofilm formation, which is also stronger in haploids [12]. There is presumably also a connection to the ploidy specificity of filamentous growth [58]. Pseudohyphal growth is a behavior of diploids starved for nitrogen, whereas the similar haploid invasive growth is induced by fermentable carbon limitation [4].

The crosses we carried out using *rim15* mutants demonstrate that some of the mating type differences we observed in the Heitman Σ1278b lineage were the result of polymorphism for a loss-of-function allele in the RIM15 locus (*rim15:1216G>T*). This allele, present in the *MATα* background, was associated with a weaker CCM phenotype. However, after breaking this linkage, we still find residual CCM variation that segregates with mating type. *MATα* strains consistently exhibit a weaker version of the CCM phenotype than do matched *MATa* strains in the Heitman background, regardless of the allelic state of RIM15. We observe a similar direction of difference between *MATa* and *MATα* in the Fink Σ1278b lineages.

### FLO11 Is Necessary for Complex Colony Formation

The flocculin Flo11p is known to be involved in several developmental processes, including filamentous growth [59] and biofilm formation [12]. There is a great deal of previous evidence of a role for FLO11 in colony morphology. For example, FLO11 was shown to be required for the “wrinkled” colony morphology observed in Ira- mutants [51], and insertion of a wild “flor” allele of FLO11 into a laboratory-domesticated strain induces the formation of “compact fluffy colonies” [35]. Finally, FLO11 is expressed at higher levels in a strain with complex morphology than a strain with simple morphology, but at very low levels in both [16]. Our finding that haploid *flo11Δ* strains fail to form complex colonies is consistent with these observations. The key stimuli we identify here, glucose and nitrogen, are both known to influence the expression of FLO11 [59],[60]. However, high levels of FLO11 expression are clearly not the sole determinant of colony morphology, since FLO11 is upregulated in diploid cells grown on SLAD (low nitrogen, high glucose) [59]. Growth on SLAD triggers pseudohyphal growth, but not the complex colony response.

### The Role of Rim15 in Complex Colony Formation

Rim15p is a protein kinase that is thought to play a central role in the integration of nutrient signals [39]. RIM15 was first identified in a screen for mutants defective in the expression of genes expressed early in meiosis [61]. Subsequent studies [40],[62] have demonstrated that Rim15p helps to regulate entry into G_0_ (stationary phase) in response to nutrient depletion, particularly glucose, by regulating the expression of a large number of stress responsive genes. Current models [39],[63],[64] posit that Rim15p integrates signals from at least three major nutrient signaling pathways, the Ras-cAMP-PKA, Sch9, and TOR pathways. Rim15-dependent effects on transcription are mediated by the transcription factors Msn2, Msn4, and Gis1 [65].

We identified and analyzed a loss-of-function mutation in RIM15 (*rim15:1216G>T*) that contributes to variation in colony morphology phenotypes among lineages of the laboratory strain Σ1278b. Our results support a model of genetic interactions in which RIM15 mutations have a modest effect on colony morphology by themselves, but can exhibit significant epistatic interactions in combination with mutations at other loci. The SNP we observed is also a strong candidate as a contributor to subtle colony morphology differences between the Heitman Σ1278b lineage and the Sigma2000 lineage. This mutation may also contribute to differences in related developmental responses, such as pseudohyphal growth, that have been noted by other investigators [27].

Since glucose limitation causes hyperphosphorylation and nuclear accumulation of Rim15p [62], and glucose limitation is also a strong inducer of complex colony morphology, we hypothesize that the CCM defects we observe in RIM15 mutants are due to a failure to trigger the upregulation of stress responsive genes via Gis1 and Msn2/4. However, the mutant phenotypes also point to the existence of one or more RIM15 independent pathways, since RIM15 mutants do not show a complete loss of colony morphology, even when combined with knockouts at other loci. One possibility is that FLO11 expression is necessary but not sufficient to induce robust colony morphology, and that Rim15p signaling might be needed to amplify or intensify the strength of the CCM response in a FLO11 independent manner.

### Outstanding Questions and Future Directions

What role, if any, does the complex colony response play in yeast ecology? It has been proposed that complex morphologies help to protect against a hostile environment [17], and the observation that some strains switch to simple morphologies after a small number of passages on rich media (i.e. auspicious conditions) may support this hypothesis [16]. It has been observed that starvation results in reorganization of yeast colonies at the cellular level [18], and there is evidence that budding patterns and distributions of cell shape are different in complex colonies than simple colonies [19]. Extensive extracellular matrix is produced by complex colonies, and is absent from simple colonies [16]. The role that we demonstrate here for RIM15 in mediating colony morphology helps to more clearly link colony morphology to stressors such as oxidative stress [65] and calorie restriction [64], where Rim15p plays an important role in mounting transcriptional responses.

Colony morphology is a phenotype that is ripe for further research. The work presented herein provides a foundation, in terms of signals and pathways, for future studies of the developmental circuitry underlying the complex colony response. While we have found important genetic intersections between colony morphology and other developmental pathways, there is clearly not a complete overlap. We found no clear change in colony morphology in many of the knockout strains we tested that are known to have altered filamentous growth. Conversely, we have identified a number of genes, such as RRT12 and RIM15, that are known to affect sporulation, but have never been shown to have filamentous growth phenotypes.

The key cellular factors that contribute to the morphogenesis of complex colonies are largely undefined. Factors such as strength of adhesion, bud location, cell shape, spatially and temporally variable rates of cell division and cell death, secretion of extracellular matrix, and other such variables must contribute in some way to establishing and maintaining colony architecture during colony growth. Future studies that exploit genetic variation among strains along with mutants and cellular reporters will help to unravel this fascinating morphogenetic process.

### Conclusion

Complex colony morphology, together with mating, filamentous growth, biofilm formation and sporulation, represent outputs of a complex decision-making machinery that integrates information on internal cell state, nutrients, potential mating partners, and various environmental stresses. A major challenge moving forward will be to better understand how simple eukaryotes such as yeast are able to correctly discriminate between different combinations of signals and how they are able to generate a diversity of responses given that the same core signaling pathways are used in different contexts.

## Materials and Methods

### Media

YEPD and Hartwell's Complete (HC) media, were made as described in Burke et al. (2000). YPD+G418 and YPD+G418+HygB contained 200mg/L geneticin. YPD+HygB and YPD+G418+HygB contained 300mg/L hygromycin B. YEPGalactose, YEPSucrose, YEPAcetate, YEPEthanol, YEPIsopropanol are the same as YEPD, except with 2% of the named carbon source (e.g. galactose in YEPGalactose) substituted for 2% dextrose. Modified YEPD media were made in the same manner as YEPD with changes as noted: 1% agar YEPD; 4% agar YEPD; 0.5% yeast extract 1% peptone YEPD; 2% yeast extract 4% peptone YEPD; 4% dextrose YEPD; 1% dextrose YEPD; 0.5% dextrose YEPD (YEPLD); 0.25% dextrose YEPD; 0.125% dextrose YEPD; 0.0625% dextrose YEPD. For “wetted” media, 400 µl of water was added to each plate and allowed to absorb; “dried” media was treated by incubation at 40C for two days. Modified synthetic complete (SC) media were made according to Kaiser et al. [66], with the following changes: 0.5% Dextrose SC (SCLD); 0.5% Dextrose SC, 50mM L-Glutamic acid monosodium salt monohydrate (SCLD+Glu); 0.5% Dextrose SC -uracil, 50mM L-Glutamic acid monosodium salt monohydrate (SCLD-Ura+Glu).

### Yeast Strains

All strains used in this work are listed in Table S2. Strains used are of diverse origin, including laboratory strains as well as clinical, distillery isolates.

To generate haploid derivatives of the homothallic diploid YJM311, the HO endonuclease was knocked out by transformation with the HO-poly-KanMX4-HO plasmid [67]. Knockouts were confirmed by PCR of the HO locus, then sporulated and tetrads were dissected. Haploid gene knockout strains PMY566, PMY568, PMY570, PMY572, PMY575, PMY577, PMY579, PMY581, PMY583, PMY585, and PMY589 were derived from diploids [26] by sporulation and tetrad dissection.

### Colony Morphology Assay

The environmental conditions tested are detailed in Table S1. Cells were plated with a targeted density of 20 or 60 cfu/plate. Several of the strains used in this study form flocs and/or aggregates of incompletely budded cell clusters. In order to accurately determine titers to plate a consistent number of cells, cultures were washed, then incubated for 15 minutes at room temperature in deflocculation buffer (90 mM mannose, 20 mM citrate, (pH 7.0), 5 mM EDTA) [68], briefly sonicated, then counted by hemocytometer. In addition to, or instead of this spreading procedure, some assays of colony morphology were conducted by pinning a small amount of yeast cells from a colony or water suspension directly to the assay plate.

For the initial survey of growth conditions, most strain-by-condition combinations were tested at two plating densities: 20 cfu/plate (results shown here) and 60 cfu/plate (data not shown). Results were similar for both plating densities. The strain-by-condition combinations not replicated are ones that showed no CCM: neither of the S288C derivatives (BY4743 and BY4739), were replicated; the wetted, dried, and room temperature conditions were also not replicated.

Once carbon limitation was determined to induce the colony morphology response, we found that YEPD with 0.5% Dextrose (Yeast Extract, Peptone, Low Dextrose - YEPLD), to be nearly optimal, strongly inducing the response while allowing sufficient colony growth to permit development of characteristic morphology (Figure 3). This medium was therefore used as a standard for subsequent experiments.

For the treatment screen, all plates were scored for colony morphology every day from day one to day six. Haploid derivatives of YJM311 were scored on day six. Mutant strains were scored on day 6 and compared to parental wild-type colonies.

### Colony Morphology Scoring

We developed a qualitative method of scoring colony morphology using a scale from zero to five, based on the complexity and definition (depth) of morphology structures. While this framework is subjective, all scoring was performed by a single individual to ensure consistency. Scores were determined based on the survey of all the colonies on a plate, rather than a single colony (although for almost all plates, the colonies on a plate all had very similar morphology). The numerical scores have the following meanings: (0) No colonies or microcolonies; (1) Simple colony morphology; (2) Hints of colony morphology; (3) Weak or early colony morphology; (4) Strong colony morphology; (5) Very strong colony morphology (Figure S8). In summary, colonies that have no signs of CCM, but have a non-smooth surface texture receive a score greater than one but less than two. Colonies that have some signs of CCM receive a score of two or greater but less than three. Colonies that have definite morphology receive a score of three or greater.

### Transposon Mediated-Mutagenesis Screen

Genome-wide transposon-mediated mutagenesis was carried out following the methods of Kumar and Snyder [69], with modifications as noted, using an mTn7 mutagenized *S. cerevisiae* genome library generated by Kumar et al. [31]. Briefly, individual pools of mutagenized library were digested with Not I to linearize, then transformed [70] into PMY574. The transformation reactions were plated onto SCLD-Ura+Glu, to simultaneously select for transformants and induce colony morphology. Colonies displaying a loss of complex morphology relative to PMY574 were picked and pinned to YEPLD to confirm the colony morphology phenotype. DNA was extracted from loss-of-morphology mutants using the DNeasy Blood & Tissue Kit (Qiagen), following the supplementary protocol for yeast DNA. Transposon insertion locations were identified by two-step PCR (ST-PCR) [71]. Primers mTn [69] and THG.SEQ [72] were used as ST-PCR primer 1 and primer 3 respectively to amplify from the “left” end of mTn7, and primers mTn7_5895R (GCACTGTTTTTATGTGTGCGATA) and mTn7_6007R (GCCGTTTACCCATACGATGT) were used as ST-PCR primer 1 and primer 3 respectively to amplify from the “right” end of mTn7. Primers 2 and 4 were as described [71]. Primers THG.SEQ and mTn7_6007R were used for sequencing ST-PCR products from the left and right ends, respectively. Finally, sequencing reads were BLASTed against the *S. cerevisiae* genome in order to locate their position within the genome.

Genes identified by mutagenesis were confirmed for colony morphology phenotypes by construction of knockout mutants in the PMY574 and PMY575 backgrounds. Primers used for gene deletion and deletion confirmation were based on primer sequences generated by the *Saccharomyces* Genome Deletion Project [73], however the UP_45 and DOWN_45 ORF specific oligonucleotides were joined with primers specific for the pRS400 plasmid series, and were used to amplify the URA3 fragment from pRS406 [74]. Transformants were selected on SC –uracil, then assayed for colony morphology phenotype by growth on YEPLD.

### Tetrad Analysis

XPY5a was crossed with XPY90α to generate diploids heterozygous for deletions at the RIM15 and TPK2 loci. MLY179α was crossed with XPY90a to generate diploids heterozygous for deletions at the RIM15 and MGA1 loci. Diploids were selected by growth on YPD+G418+HygB, then sporulated and tetrads were dissected. Segregation of the RIM15, TPK2, and MGA1 alleles was determined by assaying growth of segregants on YPD+HygB, YPD+G418, and YPD+G418 respectively. Mating type of segregants was determined by crossing with AAY1017 and AAY1018, then assaying for growth on SD. Colony morphology phenotypes of segregants were assayed by growth on YEPLD.

## Supporting Information

Figure S1 Rich nitrogen is required for induction of the complex colony response. Growth on YEPLD induces the complex colony response in the strains YJM224, YJM311, MLY40, OS17, and NKY292 but growth on, SCLD does not. Growth on (SCLD supplemented with glutamate (SCLD+Glu) recovers the complex colony response, at least partially in, most strains. Scale bar is 1 mm.
(15.34 MB TIF)

Figure S2 Several gene knockouts in the Σ1278b background cause CCM phenotypes. Compared to (A) a wild-type diploid Σ1278b strain, (B) an *ira2Δ/ira2Δ* single mutant and (C) a *tpk1Δ/tpk1Δ*, *tpk3Δ/tpk3Δ* double mutant derived from it both show a mild gain in CM, when grown on YEPLD.Compared to (D) a wild-type haploid (*MATa*) Σ1278b strain (E) *gln3Δ*, (F) *ras2Δ*, (G) *ste12Δ*, (H) *ste20Δ*, (I) *tec1Δ* and (J) *flo11Δ* single mutant strains have no CCM, while a (K) *tec1Δ*, *dig1Δ*, *dig2Δ* triple mutant and a (L) *tpk2Δ*, *tpk3Δ* double mutant have weak CCM when grown on YEPLD. Scale bar is 1 mm.
(5.72 MB TIF)

Figure S3 Some gene knockouts have different phenotypes in different lineages of Σ1278b. A (A) diploid *tpk3Δ/tpk3Δ* mutant in the “Heitman” lineage of Σ1278b has a strong gain of CCM, (B) but the same mutant in the Sigma2000 lineage has no change in CCM from WT. Some mutants in haploids of the Σ1278b background have phenotypic differences between the mating types. In (C,E,G) *MATa* strains, (C) *ste11Δ*, (E) *ste7Δ*, and (G) *tpk1Δ*, *tpk2Δ* mutants, have a complete loss of CCM, while these same mutations in (D,F,H) *MATα* strains have weak but existent CCM. The opposite mating type effect is also observed, in (I) a *MATα*, *cln1Δ* strain which has a near complete CCM loss while (J) a *MATa*, *cln1Δ* strain has stronger CCM (although decreased relative to WT). Scale bar is 1 mm.
(2.51 MB TIF)

Figure S4 The colony morphology response is induced by non-standard conditions in a *hog1Δ/hog1Δ* mutant strain. CCM is induced in a *hog1Δ/hog1Δ* mutant strain (G30076), to varying extend, by growth on media containing 0.5% dextrose, 2% dextrose, 4% dextrose, and 2% ethanol, none of which induce the response in the parental (WT/WT) strain. Scale bar is 1 mm.
(10.88 MB TIF)

Figure S5 Strength of the colony morphology response is inversely related to ploidy. Isogenic strains (other than ploidy and the MAT locus) growing on YEPLD (day six) demonstrate that intensity of CCM is inversely related to ploidy, but mating type also plays a role. (A–D) MAT homozygotes and (E–G) MAT heterozygotes in the Σ1278b background. Scale bar is 1 mm.
(2.09 MB TIF)

Figure S6 Genotype-by-environment effects on colony morphology. Colonies of strain YJM311 show distinct morphologies on different media. Non-fermentable carbon sources, (a) YEPAcetate, (b) YEPEthanol, (c) YEPIsopropanol, share a similar morphology which is close to that observed on reduced dextrose, whereas (d) YEPGalactose, (e) YEPSucrose and (f) 1% Agar YEPD (note lower magnification) have a distinct radial morphologies. Scale bar is 1 mm.
(3.29 MB TIF)

Figure S7 Colony morphology observed in survey of strains from the Saccharomyces Genome Resequencing Project. By day six, surface texture is present in (A) OS279/A on YEPLD. More signs of morphology in (B) OS259/A/A on YEPIsopropanol. Definite morphology is observed for (C) OS304/A on YEPLD (D) OS17 on YEPLD (E) OS284/A on YEPLD. Scale bar is 1 mm.
(3.29 MB TIF)

Figure S8 Colonies representative of scoring standards. (1) (MLY61 on 2% yeast extract, 4% peptone YEPD, day 3), (2) (YJM311 on YEPSucrose, day 4), (3) (OS17 on 1% dextrose YEPD, day 4), (4) (NKY292 on 2% yeast extract, 4% peptone YEPD, day 5), (5) (YJM224 on 1% dextrose YEPD, day 6). Scale bar is 1mm.
(0.88 MB TIF)

Table S1 Environmental conditions tested for inducing complex colony morphology.
(0.06 MB DOC)

Table S2 Strains used in this publication. Note: For strain background, Σ1278b [2] indicates Sigma2000 lineage of Σ1278b, Σ1278b [H] indicates the lineage of Σ1278b generated by Michael Lorenz in the lab of Joseph Heitman, and Σ1278b [F] indicates the lineage of Σ1278b used by the lab of Gerald Fink.
(0.06 MB XLS)
