# Supplementary figures and images for: Environmental and Genetic Determinants of Colony Morphology in Yeast
Source: PLoS Genet. 2010 Jan 22;6(1):e1000823. doi: 10.1371/journal.pgen.1000823 (PMC2809765; doi:10.1371/journal.pgen.1000823)

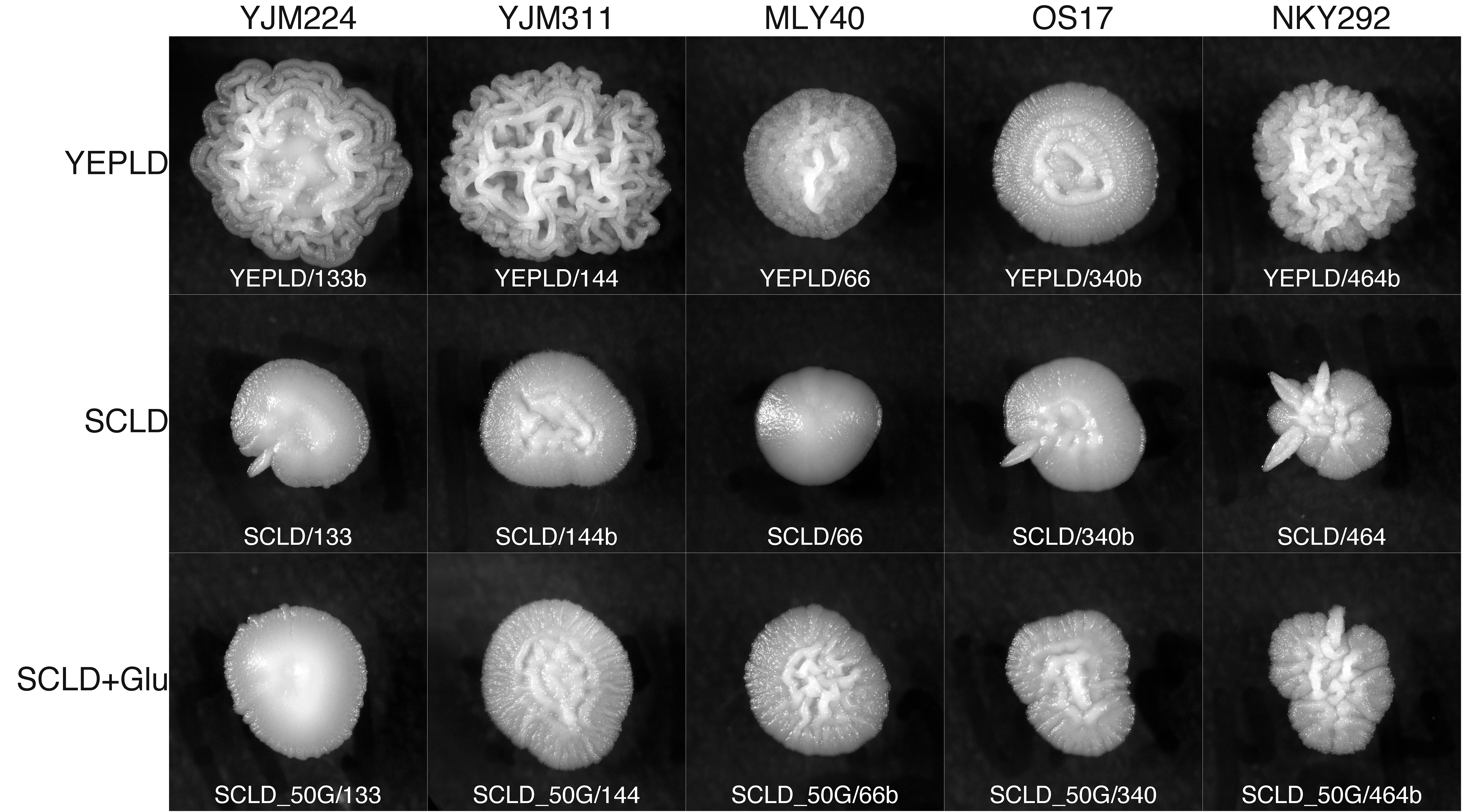

Supplement: Figure S1 — Rich nitrogen is required for induction of the complex colony response. Growth on YEPLD induces the complex colony response in the strains YJM224, YJM311, MLY40, OS17, and NKY292 but growth on, SCLD does not. Growth on (SCLD supplemented with glutamate (SCLD+Glu) recovers the complex colony response, at least partially in, most strains. Scale bar is 1 mm. (15.34 MB TIF) [file pgen.1000823.s001.tif]

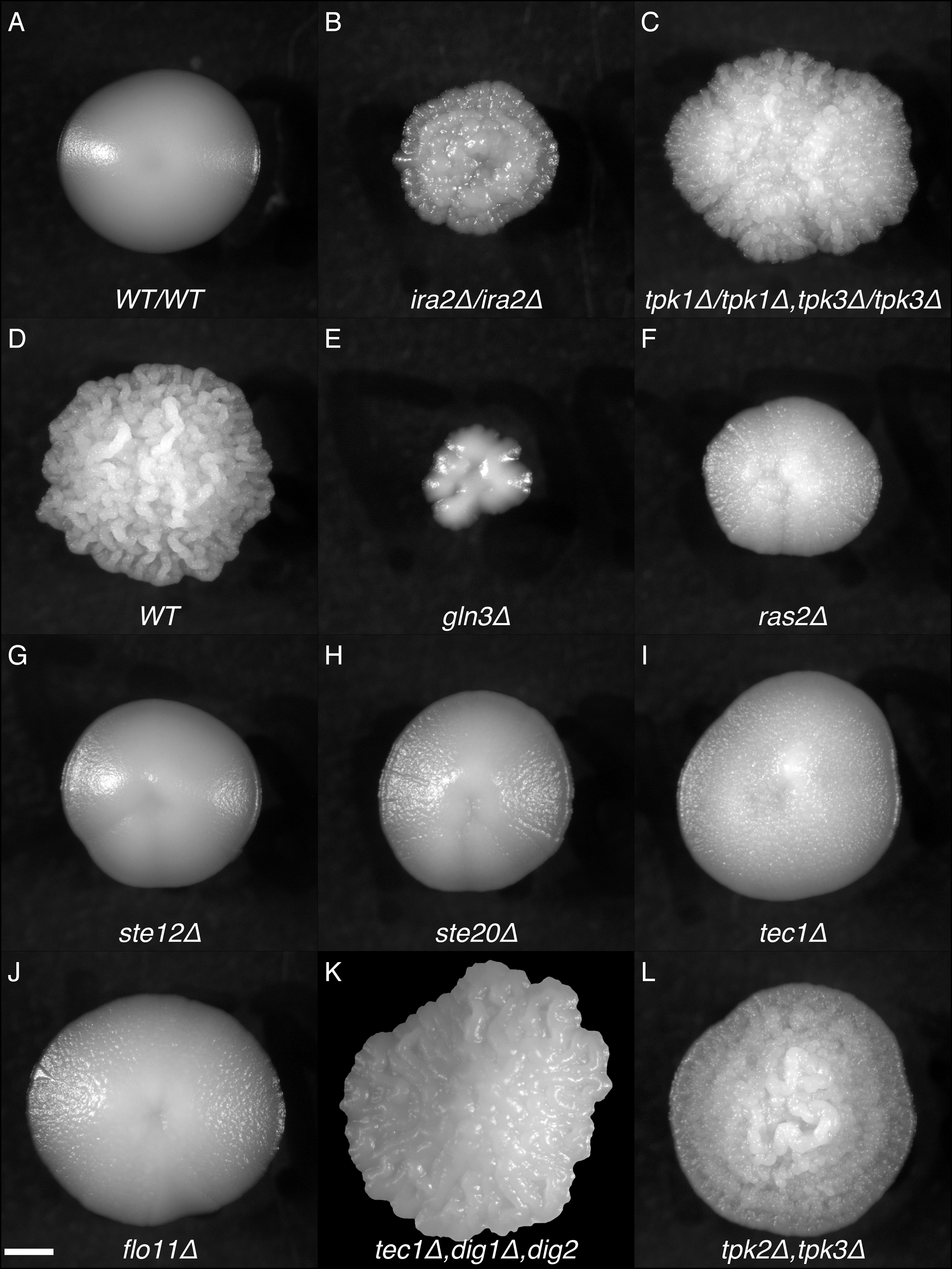

Supplement: Figure S2 — Several gene knockouts in the Σ1278b background cause CCM phenotypes. Compared to (A) a wild-type diploid Σ1278b strain, (B) an ira2Δ/ira2Δ single mutant and (C) a tpk1Δ/tpk1Δ, tpk3Δ/tpk3Δ double mutant derived from it both show a mild gain in CM, when grown on YEPLD.Compared to (D) a wild-type haploid (MATa) Σ1278b strain (E) gln3Δ, (F) ras2Δ, (G) ste12Δ, (H) ste20Δ, (I) tec1Δ and (J) flo11Δ single mutant strains have no CCM, while a (K) tec1Δ, dig1Δ, dig2Δ triple mutant and a (L) tpk2Δ, tpk3Δ double mutant have weak CCM when grown on YEPLD. Scale bar is 1 mm. (5.72 MB TIF) [file pgen.1000823.s002.tif]

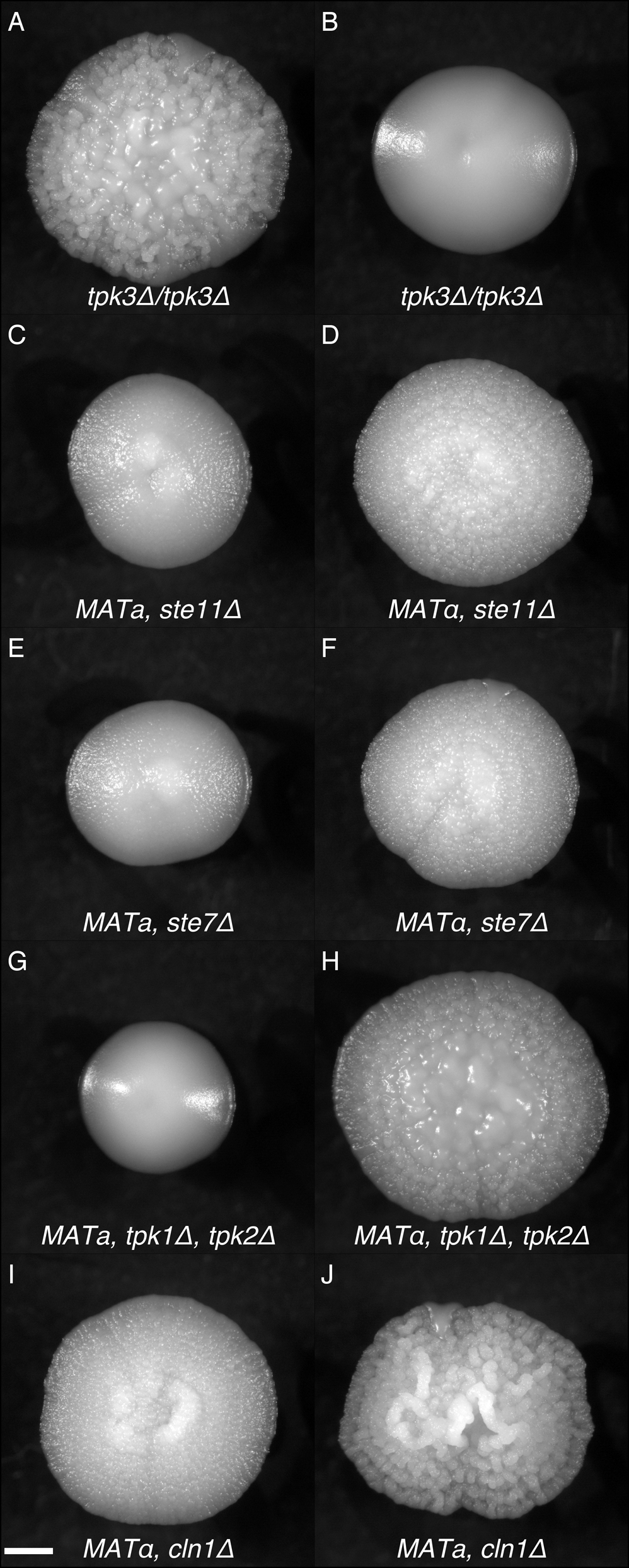

Supplement: Figure S3 — Some gene knockouts have different phenotypes in different lineages of Σ1278b. A (A) diploid tpk3Δ/tpk3Δ mutant in the “Heitman” lineage of Σ1278b has a strong gain of CCM, (B) but the same mutant in the Sigma2000 lineage has no change in CCM from WT. Some mutants in haploids of the Σ1278b background have phenotypic differences between the mating types. In (C,E,G) MATa strains, (C) ste11Δ, (E) ste7Δ, and (G) tpk1Δ, tpk2Δ mutants, have a complete loss of CCM, while these same mutations in (D,F,H) MATα strains have weak but existent CCM. The opposite mating type effect is also observed, in (I) a MATα, cln1Δ strain which has a near complete CCM loss while (J) a MATa, cln1Δ strain has stronger CCM (although decreased relative to WT). Scale bar is 1 mm. (2.51 MB TIF) [file pgen.1000823.s003.tif]

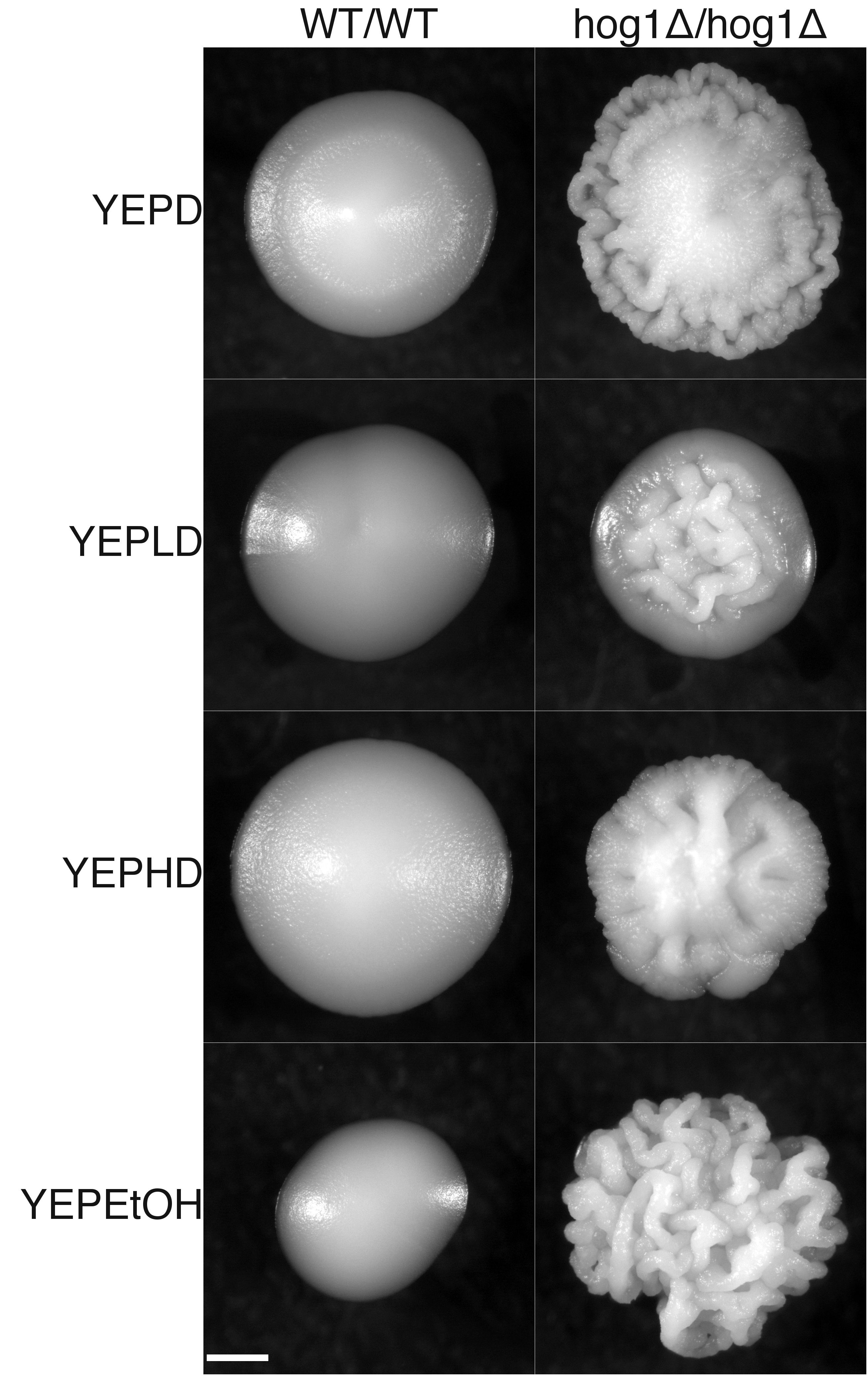

Supplement: Figure S4 — The colony morphology response is induced by non-standard conditions in a hog1Δ/hog1Δ mutant strain. CCM is induced in a hog1Δ/hog1Δ mutant strain (G30076), to varying extend, by growth on media containing 0.5% dextrose, 2% dextrose, 4% dextrose, and 2% ethanol, none of which induce the response in the parental (WT/WT) strain. Scale bar is 1 mm. (10.88 MB TIF) [file pgen.1000823.s004.tif]

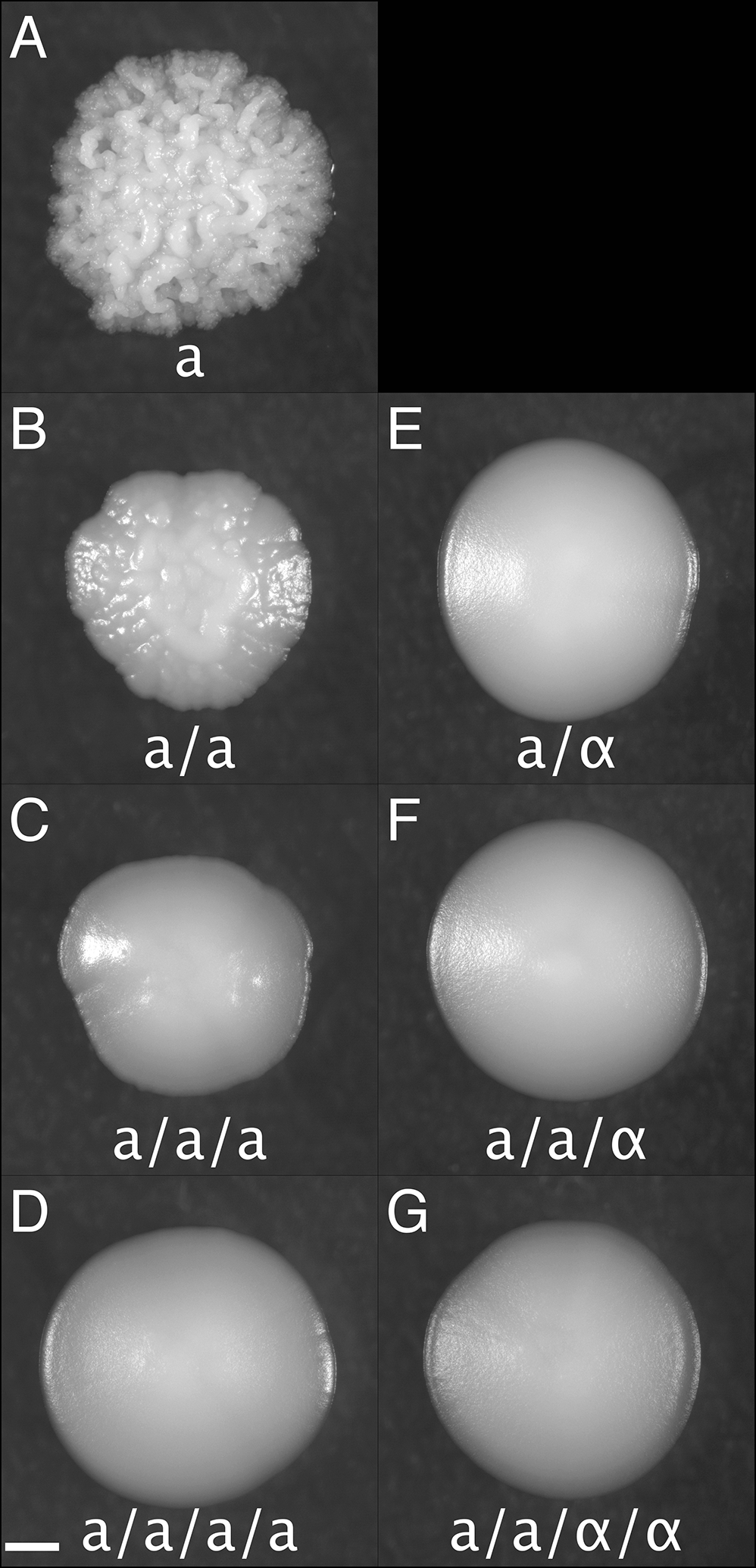

Supplement: Figure S5 — Strength of the colony morphology response is inversely related to ploidy. Isogenic strains (other than ploidy and the MAT locus) growing on YEPLD (day six) demonstrate that intensity of CCM is inversely related to ploidy, but mating type also plays a role. (A–D) MAT homozygotes and (E–G) MAT heterozygotes in the Σ1278b background. Scale bar is 1 mm. (2.09 MB TIF) [file pgen.1000823.s005.tif]

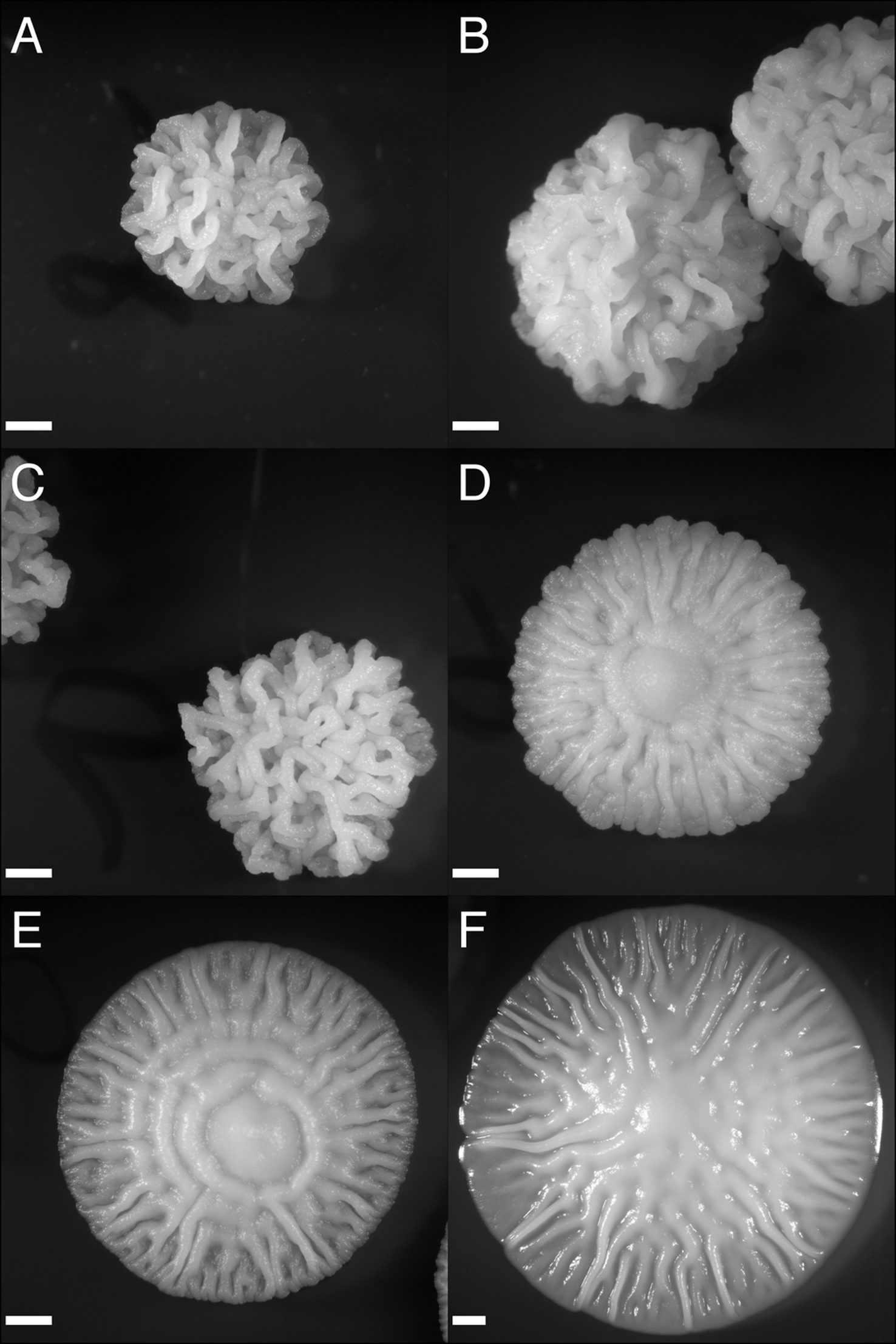

Supplement: Figure S6 — Genotype-by-environment effects on colony morphology. Colonies of strain YJM311 show distinct morphologies on different media. Non-fermentable carbon sources, (a) YEPAcetate, (b) YEPEthanol, (c) YEPIsopropanol, share a similar morphology which is close to that observed on reduced dextrose, whereas (d) YEPGalactose, (e) YEPSucrose and (f) 1% Agar YEPD (note lower magnification) have a distinct radial morphologies. Scale bar is 1 mm. (3.29 MB TIF) [file pgen.1000823.s006.tif]

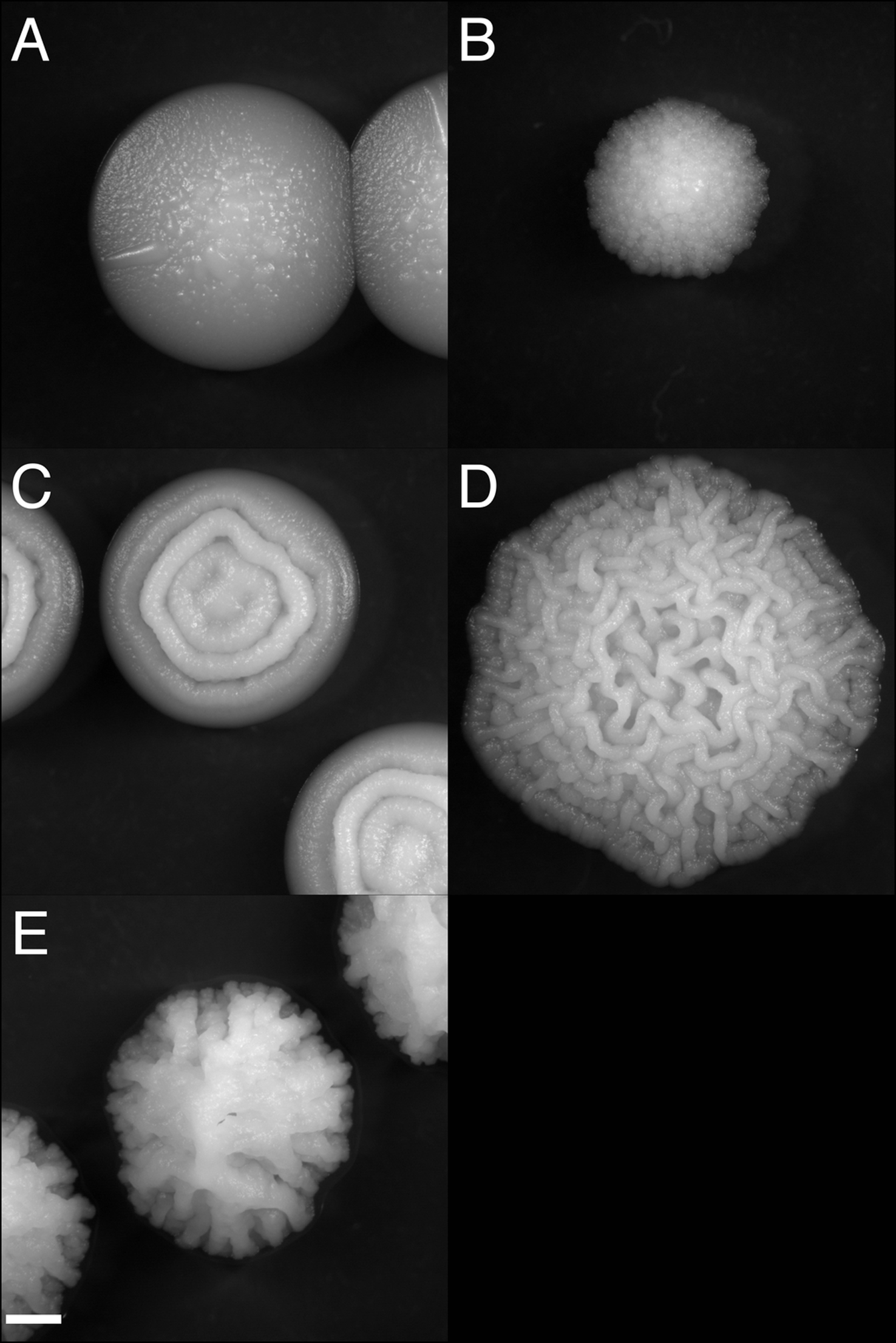

Supplement: Figure S7 — Colony morphology observed in survey of strains from the Saccharomyces Genome Resequencing Project. By day six, surface texture is present in (A) OS279/A on YEPLD. More signs of morphology in (B) OS259/A/A on YEPIsopropanol. Definite morphology is observed for (C) OS304/A on YEPLD (D) OS17 on YEPLD (E) OS284/A on YEPLD. Scale bar is 1 mm. (3.29 MB TIF) [file pgen.1000823.s007.tif]

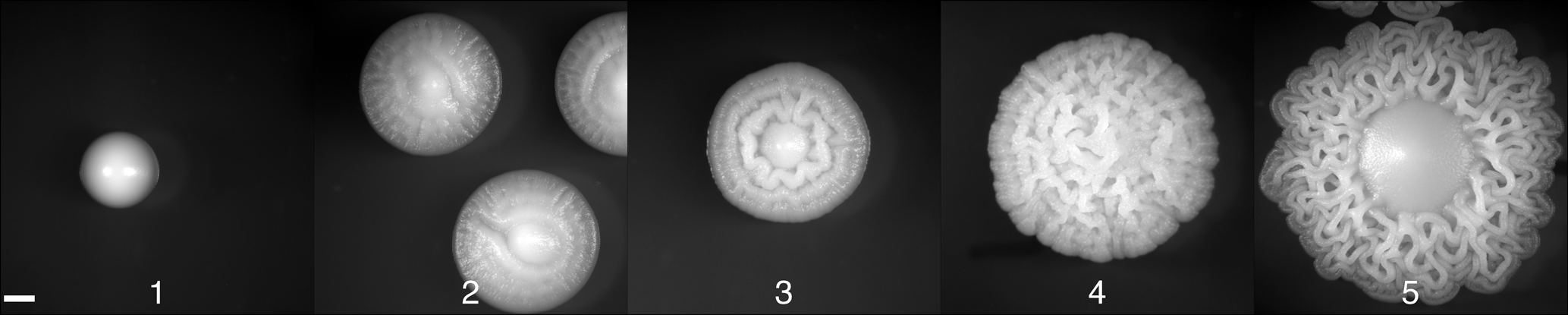

Supplement: Figure S8 — Colonies representative of scoring standards. (1) (MLY61 on 2% yeast extract, 4% peptone YEPD, day 3), (2) (YJM311 on YEPSucrose, day 4), (3) (OS17 on 1% dextrose YEPD, day 4), (4) (NKY292 on 2% yeast extract, 4% peptone YEPD, day 5), (5) (YJM224 on 1% dextrose YEPD, day 6). Scale bar is 1mm. (0.88 MB TIF) [file pgen.1000823.s008.tif]
