# Supplementary material for: Environmental and Genetic Determinants of Colony Morphology in Yeast
Source: PLoS Genet. 2010 Jan 22;6(1):e1000823. doi: 10.1371/journal.pgen.1000823 (PMC2809765; doi:10.1371/journal.pgen.1000823)
Supplement: Table S1 — Environmental conditions tested for inducing complex colony morphology. (0.06 MB DOC) [file pgen.1000823.s009.doc]

Table S1: Environmental Conditions Tested For Inducing Complex Colony Morphology

| **Media** | **Growth Temperature ( C )** | **Water Treatment** |
| --- | --- | --- |
| YEPGalactose | 30 |  |
| YEPSucrose | 30 |  |
| YEPAcetate | 30 |  |
| YEPEthanol | 30 |  |
| YEPIsopropanol | 30 |  |
| 1% agar YEPD | 30 |  |
| 4% agar YEPD | 30 |  |
| 0.5% yeast extract, 1% peptone YEPD | 30 |  |
| 2% yeast extract, 4% peptone YEPD | 30 |  |
| 1% dextrose YEPD | 30 |  |
| 4% dextrose YEPD | 30 |  |
| YEPD | 24 | Dried |
| YEPD | 24 | Wetted |
| YEPD | 24 |  |
| YEPD | 30 | Dried |
| YEPD | 30 | Wetted |
| YEPD | 30 |  |
| HC | 24 | Dried |
| HC | 24 | Wetted |
| HC | 24 |  |
| HC | 30 | Dried |
| HC | 30 | Wetted |
| HC | 30 |  |
